# Long-term efficacy of fractional microneedle radiofrequency versus botulinum toxin-A in primary axillary hyperhidrosis: a randomized controlled trial

**DOI:** 10.1007/s10103-024-04115-x

**Published:** 2024-07-10

**Authors:** Reem O. Eid, Eman Shaarawi, Rehab A. Hegazy, Vanessa Hafez

**Affiliations:** Department of Dermatology, Kasr Al-Ainy Faculty of Medicine, Cairo University, Cairo, 11956 Egypt

**Keywords:** Primary axillary hyperhidrosis, Botulinum toxin A, Fractional microneedling radiofrequency, Randomized controlled trial

## Abstract

Primary axillary hyperhidrosis is an idiopathic disorder that creates severe psycho-social burden due to excessive uncontrolled sweating. Various therapeutic agents have been described, but each has its own limitations. The use of fractional microneedling radiofrequency has emerged lately with promising results. This study aimed to determine the efficacy and safety of fractional microneedle radiofrequency in comparison to Botulinum toxin-A (BT-A) in patients with primary axillary hyperhidrosis. In this randomized controlled clinical trial, 20 patients (40 sides) were randomized to either fractional microneedle radiofrequency (4 sessions at 3-week intervals) or BT-A (single session), where each side received one of the treatment modalities. Efficacy was measured at 3, 6 and 12 months using Minor’s starch iodine test, HDSS score, Hqol questionnaire, and patient satisfaction. Fractional microneedle radiofrequency, although showed moderate efficacy, is inferior to BT-A regarding longitudinal efficacy at 12 months, as well as patients’ satisfaction. Both treatment modalities showed to be equally safe, but fractional microneedle radiofrequency procedure was substantially more painful. In conclusion, fractional microneedle radiofrequency does not offer a better substitute to BT-A in primary axillary hyperhidrosis. BT-A shows higher efficacy, is less painful, less expensive, and needs a smaller number of sessions.

## Introduction

Primary axillary hyperhidrosis is a distressing disorder that can result in skin maceration and secondary bacterial and fungal infections, in addition to appreciable social problems in both private and professional life [1]. Many therapeutic modalities have been tried for this condition, ranging from topical therapies to surgery, but each has its own limitations and side effects [2].

BT-A works by temporarily inhibiting the release of acetylcholine from the neuromascular junction; thus decreasing sweat production [3]. Onabotulinum toxin-A was FDA approved in 2004 for the treatment of axillary hyperhidrosis [4]. The average duration of symptom relief after BT-A therapy is 6 to 9 months [5, 6]. Fractional microneedling radiofrequency (FMR) is an emerging treatment modality that has been evaluated in a few studies due to its supposed potential of more permanent effect through thermal destruction of sweat glands; it is based on an oscillating electrical current that is transformed into heat energy. The direct thermal injury is transmitted from the microneedles at the interface between dermis and subcutis, causing thermolysis and decreasing in the size and density of eccrine and apocrine sweat glands [7–9].

The aim of this study was to evaluate the long term efficacy and safety of FMR in adult patients with moderate to severe primary axillary hyperhidrosis (PAH) in comparison to BT-A along 12 months.

## Methods

The protocol for this randomized controlled clinical trial (RCT) was approved by Kasr Al-Ainy Ethical Committee and was published on www.pactr.org (PACTR201808213089174). The study protocol is conformed to the ethical guidelines of the 1975 Declaration of Helsinki. Reporting of data followed the CONSORT checklist for reporting of RCTs [10].

Patients were asked to give a score for the severity of their hyperhidrosis condition according to hyperhidrosis disease severity score (HDSS) score [11]; Responses are rendered on a four-point scale: 1 = never noticeable, never interferes with daily activities; 2 = tolerable, sometimes interferes with daily activities; 3 = barely tolerable, frequently interferes with daily activities; 4 = intolerable, always interferes with daily activities.

Twenty patients older than 18 years presenting with primary axillary hyperhidrosis grades 3 and 4 HDSS and approving the study protocol were included and signed informed consent. Exclusion criteria included any history of surgery for hyperhidrosis, history of BT-A therapy within the previous 12 months, history of pacemaker implantation or any other electronic implants for incompatibility with radiofrequency apparatus, known hypersensitivity from BT-A, known neuromuscular disease (e.g. myasthenia gravis), auto immune diseases, tendency for keloid formation, and pregnancy or breastfeeding.

Patients’ axillae were randomized to receive one of both treatments using the closed envelope method, where patients were allowed to choose between two envelopes, enclosing either ‘right’ or ‘left’. The side chosen randomly by the patient was treated by BT-A, while the other side was treated by FMR. The BT-A group received a single session of BT-A intradermal injection (Refinex^®^, *Shandong Bioresearch institute, China*) using a 50IU vial injected in standard procedure [3]. The FMR group received 4 sessions at 3-week intervals using Vivace^®^ device (Shenb co., Seoul, Korea) with 3.5 mm length non-insulated needles: power Level 7, exposure time 600 msec, frequency 1 Hz. A single pass over the whole hyperhidrotic area was performed while the skin being adequately stretched, and the device handpiece firmly pressured on the skin. In both groups, the hyperhidrotic area revealed by Minors starch iodine test was marked by a surgical pen. Local anesthetic cream was applied under occlusion 30–60 min prior to the session.

Patients were assessed at baseline then after 3, 6 and 12 months from baseline for both efficacy and safety outcomes. Efficacy outcomes included the following:

1. **Investigator’s global assessment of improvement** from baseline based on photographic assessment of Minor’s starch iodine test, performed at baseline and repeated each visit till end-of-study (EOS) at 12 months. Iodine solution (5%) was applied to dry shaved axillae and after a few seconds starch was sprinkled over this area. The starch and iodine interact in the presence of sweat, leaving purplish sediment. Photographs were taken in standardized settings. The mean percent reduction from baseline of affected surface area (as seen by purplish sediment), was assessed by 2 blinded investigators on a 4-point scale: 0–25% Poor, > 25–50% Moderate, > 50–75% Good, > 75–100% Excellent.
2. **Hyperhidrosis Quality of life questionnaire (Hqol)**, performed at baseline and EOS. Patients were asked to give a score from 1 to 5 on 13 questions reflecting the effect of hyperhidrosis on the quality of their lives, where 1 indicates ‘Excellent’ and 5 indicates ‘very poor’. The questionnaire covers functional, social, personal and emotional domains, as well as special circumstances. This questionnaire is adapted from the original Hqol questionnaire designed by Campos and colleagues [12]. The total score ranges from 20 (Excellent) to 100 (very bad).
3. **HDSS score**, performed at baseline and EOS. A one-grade reduction of HDSS is equivalent to 50% reduction of sweating, and a 2-grade reduction to 80% [11, 13].
4. **Patient satisfaction** for each treated side on a 4-point scale: 0–25% Unsatisfied, > 25–50% slightly satisfied, > 50–75% moderately satisfied, > 75% very satisfied.
5. **Treatment success** at 6 and 12 months, defined as achievement of ≥ 75% reduction in Minors starch iodine test from baseline and/or ≥ 75% patient satisfaction.

Safety outcomes included monitoring of side effects as pain, bruising, infection, compensatory hyperhidrosis. Pain was evaluated on a visual analogue scale 1 to 10, where 1 is the least and 10 is the most severe pain.

Since this is the first study to be conducted on these parameters, sample size was calculated (using G*Power 3.1.9.2.) based on a pilot run of 5 patients that revealed a moderate to large effect size corresponding to Cohen’s d = 0.76, that was assumed to be clinically significant. Analysis was performed with a two tailed alpha error probability set at 0.05, the projected sample size needed for the aforementioned effect size (d = 0.76) was 17 patients to be able to reject the null hypothesis that this difference is zero with probability (power) 0.80. To account for expected 10% drop-outs and to allow for sub-group analysis, a sample of 20 patients was recruited. Data were coded and entered using the statistical package for the Social Sciences (SPSS) version 25 (IBM Corp., Armonk, NY, USA). P-values less than 0.05 were considered as statistically significant. Treatment success data were analyzed based on intention-to-treat, while other data were submitted to per protocol analysis.

## Results

Three males (15%) and 17 females (85%) were included, with mean age 27.65 ± 9.39 (range: 18 to 48 years). Baseline Hqol was 53.65 ± 6.56. Both therapeutic arms were homogenous as regards baseline HDSS (*p*-value = 0.350). 17/20 patients completed 12 months follow-up. Two patients dropped out after the 1st session, and 1 patient after his last session but he did not come for follow up.

In comparison to baseline HDSS, both BT-A and FMR groups showed a statistically significant difference of HDSS at 3, 6 and 12 months (Table 1).

**Table 1:** Comparison between baseline HDSS and HDSS at 3, 6 and 12 months (M3, M6 and M12) in BT-A group and FMR group

BT-A group	Mean ± SD	95%CI	*p* value compared to HDSS at baseline
HDSS at baseline (*N* = 20)	3.50 ± 0.51		
HDSS at M3 (*N* = 17)	1.41 ± 0.51	1.15–1.67	**< 0.001**
HDSS at M6 (*N* = 17)	1.76 ± 0.56	1.48–2.05	**< 0.001**
HDSS at M12 (*N* = 17)	2.47 ± 0.51	2.21–2.74	**< 0.001**
FMR group			
HDSS at baseline (*N* = 20)	3.65 ± 0.49		
HDSS at M3 (*N* = 17)	2.12 ± 0.49	1.87–2.37	**< 0.001**
HDSS at M6 (*N* = 17)	2.59 ± 0.62	2.27–2.91	**< 0.001**
HDSS at M12 (*N* = 17)	3.18 ± 0.53	2.90–3.45	**0.002**

BT-A: Botulinum toxin A, FMR: fractional microneedle radiofrequency, HDSS: hyperhidrosis disease severity scale, CI: confidence interval

**Fig. 1:**
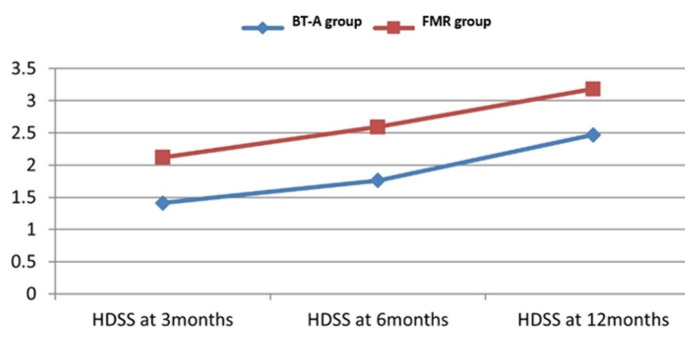
Line chart showing uprising mean hyperhidrosis disease severity scale (HDSS) in both groups at 3, 6 and 12 months, with less hyperhidrosis severity (less HDSS) in botulinum toxin (BT-A) group in comparison to fractional microneedle radiofrequency (FMR) group all through

**Fig. 2:**
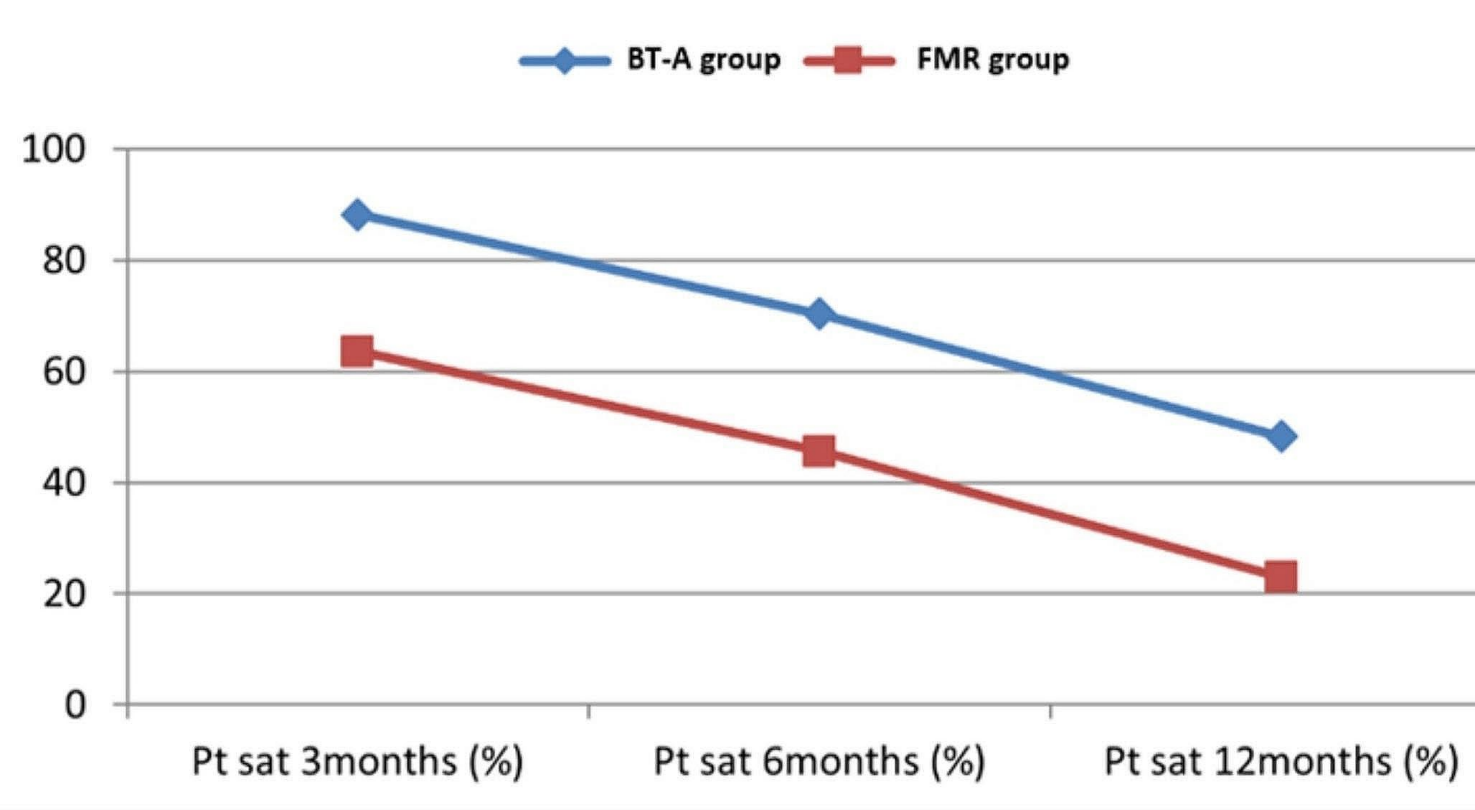
Line chart showing a declining mean percent patient satisfaction (Pt sat) of both groups at 3, 6 then 12 months, with constant superior satisfaction in botulinum toxin (BT-A) group in comparison to fractional microneedle radiofrequency (FMR) group

**Fig. 3:**
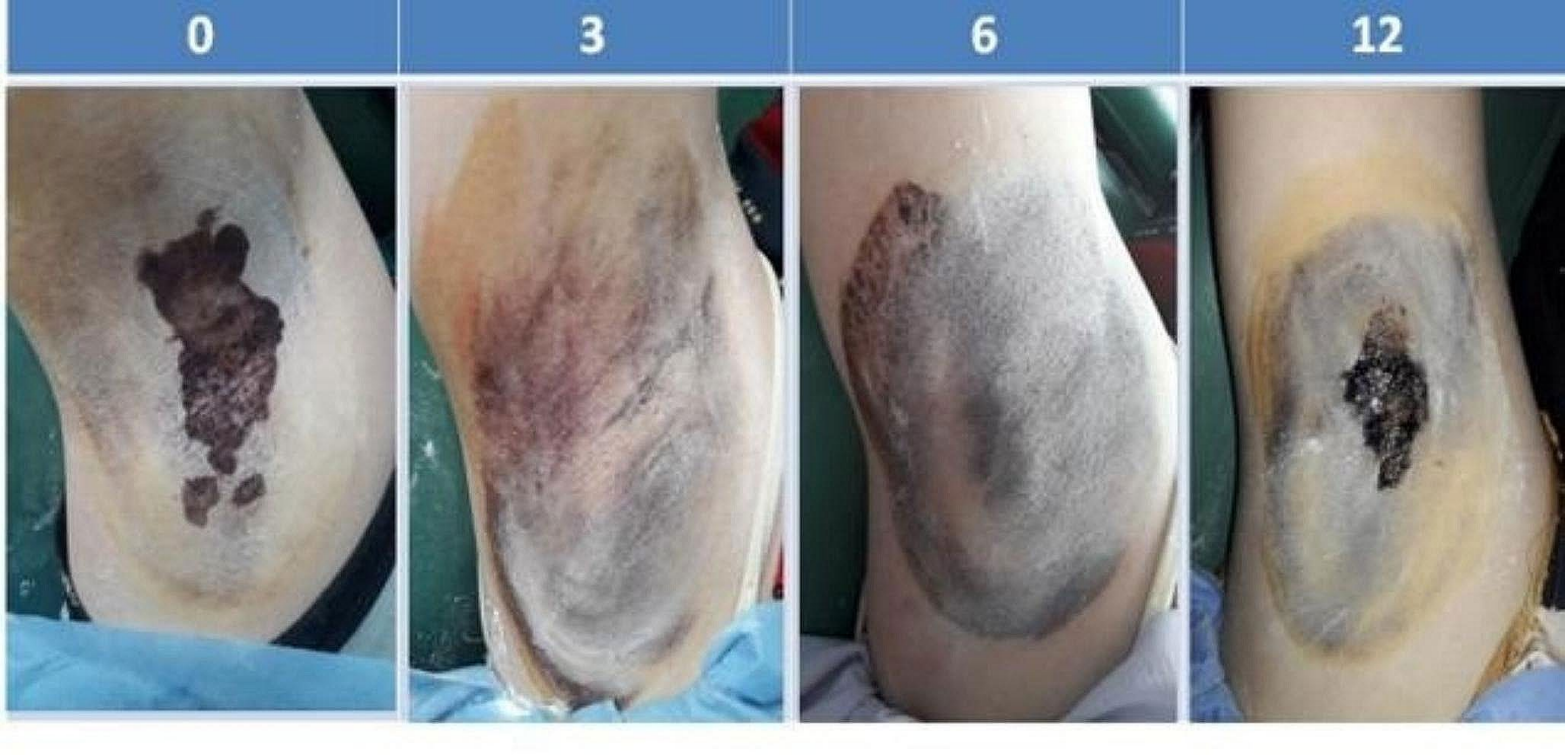
Minor’s starch iodine test in a side treated by fractional microneedling radiofrequency: Excellent response at 3 and 6 months, 50% relapse at 12 months

At 3, 6 and 12 months, BT-A was found significantly more effective than FMR as demonstrated by a significantly lower mean HDSS score, lower mean Hqol, and higher mean percent patient satisfaction in comparison to baseline (Table 2). Figures 1 and 2 demonstrate the gradual decline of efficacy of both interventions and the superior efficacy of BT-A all through 12 months. Figure 3 shows starch iodine test in a representative side treated with FMR.

**Table 2:** Comparative efficacy outcomes of BT-A versus FMR at 3, 6 and 12 months (M3, M6, M12)

		BT-A group (*N* = 17)	FMR group (*N* = 17)	*P* value
MSIT percent reduction M3	Mean ± SD	88.68 ± 10.0	83.97 ± 12.06	0.241
	95% CI	83.7-94.28	77.77–90.17	
HDSS M3	Mean ± SD	1.41 ± 0.51	2.1 ± 0.49	**< 0.001**
	95% CI	1.15–1.67	1.87–2.37	
Hqol M3	Mean ± SD	17.41 ± 4.43	24.94 ± 5.54	**< 0.001**
	95% CI	15.13–19.69	22.09–27.79	
Patient satisfaction M3	Mean ± SD	88.24 ± 10.89	63.53 ± 16.84	**< 0.001**
	95% CI	82.64–93.83	54.87–72.19	
MSIT percent reduction M6	Mean ± SD	84.12 ± 14.50	68.24 ± 16.55	**0.006**
	95% CI	76.66–91.54	59.72–76.75	
HDSS M6	Mean ± SD	1.76 ± 0.56	2.59v0.62	**< 0.001**
	95% CI	1.48–2.05	2.27–2.91	
Hqol M6	Mean ± SD	22.00 ± 4.26	28.76 ± 5.36	**< 0.001**
	95% CI	19.81–24.19	26.01–31.52	
Patient satisfaction M6	Mean ± SD	70.29 ± 18.75	45.59 ± 20.15	**0.001**
	95% CI	60.66–79.93	35.23–55.95	
MSIT percent reduction M12	Mean ± SD	63.38 ± 12.68	49.41 ± 16.07	**0.008**
	95% CI	56.86–69.90	41.15–57.67	
HDSS M12	Mean ± SD	2.47 ± 0.51	3.18 ± 0.53	**< 0.001**
	95% CI	2.21–2.74	2.90–3.45	
Hqol M12	Mean ± SD	29.82 ± 5.04	35.35 ± 5.12	**0.003**
	95% CI	27.23–32.42	32.72–37.99	
Patient satisfaction M12	Mean ± SD	48.24 ± 18.45	22.94 ± 20.31	**0.001**
	95% CI	38.75–57.72	12.50-33.39	

BT-A: Botulinum toxin A, FMR: Fractional microneedle radiofrequency, MSIT: Minor’s starch iodine test, HDSS: Hyperhidrosis disease severity scale, Hqol: Hyperhidrosis quality of life, CI: Confidence interval

Treatment success at 6 months was significantly better in BT-A versus FMR group: 15/20 (75%) and 6/20 (30%) respectively (*p*-value = 0.004). At 12 months, the significant difference was lost: 3/20 (15%) and 1/20 (5%) respectively achieved treatment success (*p*-value = 0.605).

Patients reported significantly higher mean pain score in FMR versus BT-A group (6.48 ± 0.93, 95% CI: 6.04–6.91 versus 4.00 ± 1.75, 95% CI: 3.18–4.82, *p* < 0.001). Compensatory hyperhidrosis manifested in 2 patients on the same side treated with BT-A. There was no bruising, infection or post-inflammatory pigmentation recorded in any group. One patient complained of itching on the side treated with FMR.

No significant correlation was found between age, baseline HDSS or HqoL and any of the outcomes.

## Discussion

The results of this long-term study show that a single session of BT-A injection is more effective in management of PAH and yield higher patient satisfaction than four sessions of FMR, with the microneedle type and parameters used in the present study. Both interventions show best efficacy after 3 months, then a gradual decline is observed, although some efficacy is still preserved in a few patients up to 12 months. This study is the first to compare both procedures in a longitudinal follow-up for 12 months. Both treatment modalities were shown to be equally safe, but FMR procedure was more painful compared to BT-A injection.

The long-term maintenance of efficacy was demonstrated by a statistically significant difference between HDSS at baseline and HDSS at 3, 6 and 12 months in both BT-A and FMR groups. However, BT-A showed significantly higher treatment success at 6 months in comparison to FMR (75% of patients in BT-A group versus 30% of patients in FMR group). It seems that both procedures peak at 3 months, but the decline of efficacy is faster on the FMR side, reaching minimal efficacy at 6 months in comparison to BT-A. We defined treatment success based on a patient-oriented satisfactory success so as to ensure a realistic outcome.

The higher efficacy of BT-A was evidenced at 3, 6 and 12 months by a significantly lower mean HDSS score, lower mean Hqol (better quality of life), and higher mean percent patient satisfaction. Moreover, a significantly higher mean percent reduction of MSIT was recorded at 6 and 12 months in the BT-A group.

The superiority of BT-A over two sessions of FMR was previously reported in a three-months split-body comparative study in Thailand [14]. At 3-months, BT-A demonstrated significantly lower HDSS, better quality of life (measured with DLQI), higher patient satisfaction and less transdermal water loss. Long-term efficacy was not studied.

The efficacy and safety of FMR was previously assessed in several studies with average results and high safety profile, as in the present study [7–9, 14, 15]. Of note, most of treated patients were females, as in our study, denoting a higher distress and urge for treatment in this population [8, 14]. All the studies but one [7] applied 3 treatment sessions to their patients, compared to 4 sessions in the current study. While most of studies did not clarify, the use of insulated needles at 3 depths (2, 3 and 3.5 mms) was previously reported in one study [15] in contrast with our work where non-insulated needles were used at 3.5 mm depth, and the results are comparable (80% of patients achieved ≥ 50% reduction in sweat production at 6 months, versus 70.5% of our study patients). This suggests that the use of non-insulated needles in a single-pass method, first applied in the current work, might offer a more practical alternative to the several passes method with insulated needles, minding a higher pain with non-insulated needles. Nevertheless, the long-term effectiveness of FMR reported by a sham-controlled study from Iran [9] is better than the one we achieved (mean HDSS score at 12 months 2.5 versus 3.18, respectively). This difference might be attributed to different parameters used, operator-dependent factors, or genetic susceptibility among different ethnic populations. The use of insulated needles in the latter study with 3 passes targeting 3 different depths might account for the better long-term efficacy demonstrated, due to cumulative destruction of sweat glands by the delivered thermal energy.

The depth of sweat glands, both eccrine and apocrine, was evaluated in the axilla using post-mortem histological analysis [16] and ultrahigh frequency ultrasound in Japanese patients [16, 17]. The secretory coils were found located at the boundary between dermis and subcutaneous tissue, and the vertical depth ranged from 1.5 to 3 mm. Therefore, the use of FMR needles with full length (3.5 mm) and good coaptation of the handpiece with the stretched skin should deliver the necessary energy to the full length of the sweat glands. But it seems that the regenerative power of the glands or the lack of adequate power might compromise the permanent efficacy of FMR. This aspect warrants further investigation with clinicopathological study of the effects of FMR on the sweat glands destruction. Unfortunately, we could not obtain patient consent for skin biopsies to study the histological response to treatment in this work.

It is of interest that we observed a better result achieved by thin patients than overweight patients receiving FMR treatment (based on clinical observation). A significant correlation between HDSS change and body mass index was previously documented after FMR sessions [9]. Also of interest, the skin tightness noticed after FMR sessions contributed to patient satisfaction in one of our patients, emphasizing the role of FMR in cosmesis. Previously reported adverse events include pain and hyperpigmentation [7]. The latter did not occur in our hands.

The results of the non-comparative studies conducted on BT-A came consistent with the current study as regards outcomes, safety and patient satisfaction for BT-A in hyperhidrosis. [5, 18–23] Most of the studies applied similar techniques to the current work. The dose of BT-A ranged from 50 IU like in this study to 100 IU [22] and 200 IU [18]. Quantitative assessment of effectiveness was additionally performed in some studies using gravimetry [18, 20, 23]. Similar to our results, a significant effectiveness was achieved by BT-A at 3 months, as evidenced by a mean reduction of 2 points in HDSS [21], in approximation to our 3-months results (mean HDSS score reduced from 3.50 to 1.41). Mild and transient side effects were reported in 6% [22] and 11% [19] of patients, namely compensatory hyperhidrosis which was similarly manifested by 11.8% of our study patients on the same treated side.

## Conclusions

Fractional microneedle radiofrequency does not offer a better substitute to BT-A in primary axillary hyperhidrosis, even if the number of sessions is increased to four sessions. BT-A shows better efficacy up to 12 months, is less painful, less expensive, and needs less number of sessions. The results of a single-pass technique using non-insulated needles for FMR, as in this work, gives comparable results to previous studies using multiple pass method with insulated needles, but with less prolonged efficacy.
